# Participatory Communication and Mass Dog Vaccination for Effective Rabies Management in Mitooma District, Western Uganda: An Exploratory Study

**DOI:** 10.1002/puh2.70399

**Published:** 2026-09-29

**Authors:** Augustino Ngabirano, Matthew Chibunna Igwe, Charles Idehen

**Affiliations:** ^1^ Department of Public Health School of Allied Health Sciences Kampala International University—Western Campus Kampala Uganda; ^2^ Department of Production—Field Extension Department of Production—Agricultural Field Extension Mitooma District Local Government Bushenyi Uganda

**Keywords:** human–dog–wildlife interface, mass dog vaccination, Mitooma District, One Health, participatory communication, rabies control

## Abstract

**Background:**

Canine rabies remains an endemic public health threat in Mitooma District, Uganda, where traditional, top–down public health interventions frequently fail to achieve the required 70% mass dog vaccination (MDV) coverage due to limited community engagement and one‐way communication.

**Objectives:**

This exploratory study evaluated the effectiveness of participatory communication in enhancing community engagement in MDV programs at the human–dog–wildlife interface in Mitooma District and identified the sociocultural and ecological factors influencing rabies control.

**Methods:**

Four gender‐segregated focus group discussions were conducted across four purposively selected parishes (Kiyanga, Kaburara, Iraramira, and Kashasha) in Kiyanga sub‐county, Mitooma District. Data were analyzed using thematic framework analysis.

**Results:**

Critical gaps persist regarding paralytic rabies, non‐bite transmission pathways, and wildlife reservoirs, whereas inadequate interactive education drives the adoption of harmful traditional wound care. Furthermore, top–down vaccination mismatches and utilitarian canine views trigger booster noncompliance and ownership denial to evade medical and legal liabilities.

**Conclusion:**

Rabies persistence in Mitooma District is fundamentally driven by a failure of bidirectional community engagement and multi‐sectoral coordination. Shifting from top–down public health messaging to a One Health participatory communication framework is necessary to establish sustainable community co‐ownership and break the transmission cycle.

## Graphical abstract

This study evaluates participatory communication strategies in mass dog vaccination campaigns within Mitooma District. By actively engaging local communities, the initiative significantly enhanced vaccination coverage, improved public awareness of rabies prevention, and established a scalable, community‐driven framework for effective zoonotic disease management in rural, resource‐limited settings.

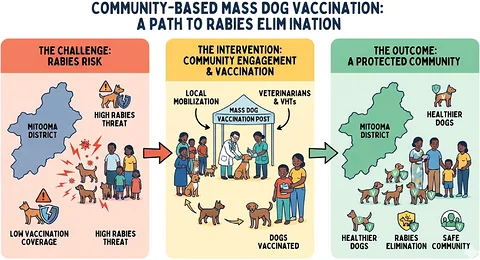

## 1 Introduction

Rabies remains a significant public health threat in Uganda, where mass dog vaccination (MDV) efforts are hindered by limited community participation and a historical reliance on top–down approaches. Implementing participatory communication strategies is essential to foster community engagement and achieve the World Health Organization's targeted 70% vaccination coverage. This article fills a gap by giving results from an exploratory study conducted during a mass dog rabies vaccination program in Mitooma District, western Uganda, in March 2024. As a fatal zoonotic disease, its common route of transmission to humans is through untreated dog bites from infected animals [1, 2]. With less than 10% vaccination coverage of Uganda's pets, the country still reports a high incidence of rabies with rural areas disproportionately affected, highlighting the country's stark immunization gaps [3]. The high incidence of rabies in Uganda may be attributed to a number of factors, including the geo‐climatic seasonal variations [4] and a high amount of greenery that serves as a haven for stray dogs, yet a formalized mechanism for monitoring the rabies epidemic is absent in the country [5]. To prevent canine‐mediated rabies, the Ugandan government conducts yearly dog vaccination and rabies monitoring programs throughout all districts.

The Ministry of Agriculture, Animal Industry and Fisheries (MAAIF) oversees these initiatives through the Veterinary Department [6, 7]. However, low community engagement and participation hinder program effectiveness, making it difficult to reach the World Health Organization's 70% vaccine coverage target [8] given that the vaccination initiatives have mostly taken a top–down approach, and not effectively educating the public about responsible dog ownership or changing their perception to take stray dogs and wildlife as a community obligation [9]. To enhance and heighten this initiative, participatory communication, a strategy that has been successfully applied in various health contexts to enhance community involvement, has been advocated seeking to enlighten, educate, and modify behavior and ultimately contributing to more equitable and sustainable mass vaccination program for the dogs [10], but its application in rabies control at the human–dog–wildlife interface is understudied, and its effectiveness in this context is unknown. This vaccine‐preventable disease is endemic in many countries where domestic dogs serve as the principal reservoir for the rabies virus that accounts for as high as 99% of human exposures and fatalities [1, 2].

The disease is predominantly high in low‐resource settings, majorly affecting children and the elderly [11], and the disease is nearly always lethal once clinical symptoms appear [1, 2]. It spreads through contact with bite wounds, open skin cuts, and mucous membranes from saliva or other infectious materials like brain tissue [12]. It is estimated that of all rabies deaths globally, 95% are thought to occur in Asia and Africa combined, whereby 21,476 human deaths from dog‐mediated rabies occur in Africa alone annually [1, 13]. Despite the fact that vaccination rates for dogs have significantly increased in Africa [14], there are still issues with resource mobilization as well as operational and cultural hurdles that need to be properly understood and dealt with in the appropriate context [15, 16]. Consequent to the numerous public awareness campaigns [17], these days, the majority of people living in developing nations are largely aware that rabies exists [18], and in certain areas, their knowledge of the disease's major preventative and exposure risks has improved [18, 19]. Nonetheless, attitudes and behaviors of general rabies control are still impacted by significant information gaps about dog bite prevention techniques and the proper way to respond to such incidents [20, 21].

Significantly underreported as a result of inadequate surveillance and laboratory infrastructure [22], inadequate field‐based testing protocols as well as misdiagnosis [23, 24], rabies affects local communities, public health sector budgets, and livestock industries in the world's poorest regions, with an estimated $8.6 billion in economic losses each year [25]. Through widespread dog vaccination campaigns, rabies in both humans and dogs can be managed and occasionally eradicated in developing nations. Rabies elimination and attaining the zero‐by‐30 strategy rely on countries stepping up their dog vaccination programs to the target of 70% coverage as the critical directly measurable indicator on this commitment [26]. As the most economical approach now available, the latest Global Strategy Plan to halt human mortality from dog‐mediated rabies by 2030 recommends widespread dog vaccination campaigns spread over several years [27]. It is expected that during yearly vaccination programs, at least 70% of dogs should receive vaccinations to prevent rabies [1]. An understanding of the dynamics of dog ownership, the interaction between humans and dogs, and the ecology of the dog population—all of which might differ depending on the location or be influenced by sociocultural factors [28] is generally helpful in directing long‐term rabies control initiatives in particular areas [29].

Effective rabies management efforts require an awareness of the roles that many species play in the transmission of multi‐host viruses [13]. Although all mammals are rabies‐prone, some species are more likely than others to contract and transmit it to others [13, 30]. Therefore, rabies control strategies could be effective if we have a comprehensive grasp of the species that are necessary to keep the rabies virus circulating (the maintenance hosts) [31]. Across Africa and Asia, domestic dogs are thought to be the principal maintenance hosts for rabies, and dog bites cause more than 99% of human rabies fatalities [13, 32]. Even though it has been repeatedly shown that rabies vaccinations for domestic dogs are both effective and affordable in preventing human rabies, dog vaccination programs are still not generally adopted in many countries where rabies is endemic [33, 34]. The main cause of this is a lack of funding for canine vaccinations, although worries that wildlife could contribute to the spread of the disease and make vaccinations ineffective are frequently voiced [35, 36]. Nevertheless, domestic dog vaccination decreased the risk to humans even in locations with a comparatively high percentage of wildlife cases [37, 38]. The significance of continuing annual dog vaccination programs is observed in the rise of canine rabies cases after vaccination campaigns have ended [39].

This increase in dog rabies and the probable subsequent rise in wildlife rabies pose a serious risk to the public health [40]. Over the years, numerous methods have been tried globally to eradicate rabies in both humans and canines [41], such as mass dog euthanasia [42] or using conventional treatment to treat bite wounds [43]. According to available data, maintaining dog vaccination and managing the dog population while integrating the community is one strategy that must work in tandem to avoid rabies in people [9]. In light of this, the majority of modern rabies extermination plans adhere to the “One Health” concept, which adopts a sociocultural strategy that includes MDV campaigns, public outreach and education campaigns, government advocacy, and humane dog population control measures [44]. Mitooma District is presently focusing its rabies elimination tactics on vaccination and managing stray dog populations because research indicates that “unvaccinated owned, stray or community dogs are the major reservoirs of rabies worldwide” [45]. In light of the fact that dog bites account [46] for almost all human rabies transmissions, optimizing localized communication to drive program uptake remains vital.

## 2 Materials and Methods

This exploratory, qualitative descriptive case study was conducted in March 2024 across four parishes (Iraramira, Kaburara, Kiyanga, and Kashasha) in Kiyanga sub‐county, Mitooma District, southwestern Uganda—a region bordering the Maramagambo Forest Reserve within the Queen Elizabeth Conservation Area (Figure 1). This setting provides a representative ecological and sociodemographic context for evaluating localized MDV efforts. The study assessed participatory communication strategies during a large‐scale dog vaccination program using focus group discussions (FGDs), informal interviews, and field observations.

**FIGURE 1:**
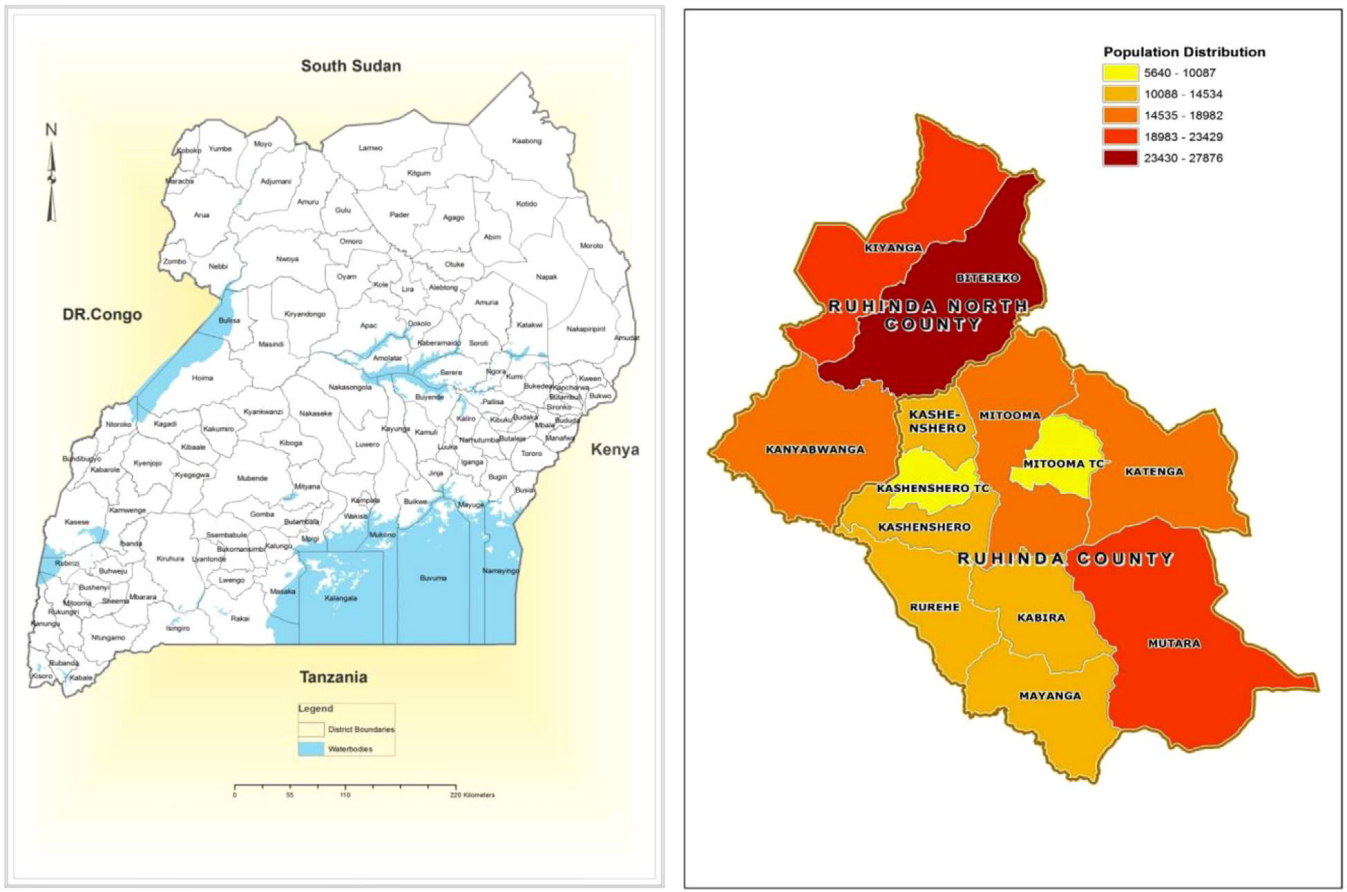
Map of Uganda showing the location of Mitooma District and Kiyanga sub‐county.

A non‐probability sampling strategy was utilized. All four constituent parishes were selected as primary vaccination sites with the assistance of the assistant animal husbandry officer. Purposive sampling was applied to select administrative stakeholders and target villages based on past rabies prevalence and campaign timing, whereas convenience sampling was used to recruit dog owners at active vaccination sites. To mitigate selection bias, data collection teams rotated morning and afternoon shifts across multiple geographic clusters to capture a broader demographic range.

Four gender‐homogeneous FGDs were conducted in the native language (Runyakitara), involving 44 participants (25 men, 19 women; Table 1). Each FGD (8–13 participants, taking approximately 40 min) followed a semi‐structured guide addressing four thematic domains: rabies literacy, management/control measures, risk behaviors, and canine societal roles. To complement and triangulate these data, the researcher conducted targeted informal discussions and immersive participant observations (Figure 2) with local council leaders, villagers, school teachers, clinical nurses, and veterinarians until theoretical data saturation was achieved.

**TABLE 1:** Features of the focus group discussions (FGDs) by parish, group type, and dog ownership.

Parish	Group type	Participants (*n*)	Dog owners	Non‐dog owners
Kiyanga	Men (FGD 1)	13	11	2
Iraramira	Women (FGD 2)	8	5	3
Kaburara	Men (FGD 3)	12	9	3
Kashasha	Women (FGD 4)	11	7	4
**Total**	**4**	**44**	**32**	**12**

**FIGURE 2:**
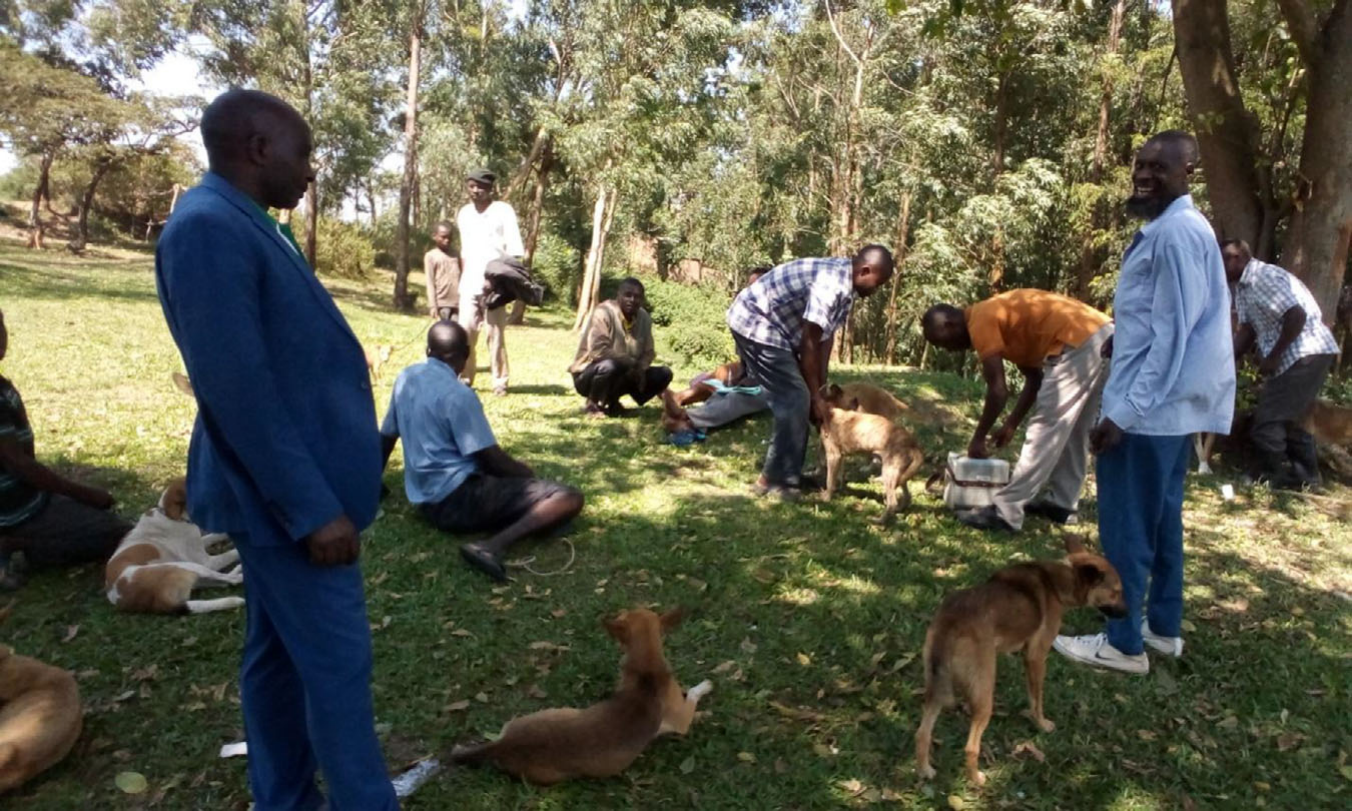
A researcher conducting targeted informal discussions and participant observations at the vaccination site.

Audio recordings from FGDs and interviews were transcribed verbatim, translated into English, and analyzed via inductive thematic analysis following Braun and Clarke's framework. Two independent researchers coded a subset of transcripts to establish a uniform codebook, resolving discrepancies through consensus. Recurrent codes were then aggregated into overarching themes regarding participatory communication facilitators and hurdles. Rigor and trustworthiness were maintained through extensive field notes for triangulation and member‐checking with selected local leaders.

This study was conducted as an institutional program evaluation of a routine mass dog rabies vaccination campaign managed and overseen by the Mitooma District Local Government Veterinary Department, which granted administrative clearance, institutional consent, and formal ethical authorization for qualitative data collection. Academic and scientific oversights were maintained in strict accordance with the lead investigator's doctoral research protocols at Kampala International University. Because this exploratory study relied entirely on qualitative methodology involving human participants (FGDs and informal interviews) regarding public health communication channels and did not involve experimental manipulations, handling, restraint, biological sampling, or clinical interventions on live animals; formal animal welfare committee protocols for invasive veterinary research were not triggered. Prior to data collection, all participants were thoroughly informed in Runyakitara about the study's objectives, data confidentiality, and the voluntary nature of their participation. All dog owners and community stakeholders provided informed verbal consent before participating.

## 3 Results

Community perceptions of rabies across Mitooma District are characterized by a profound paradox: Although a baseline awareness of the disease's fatal nature and transmission through bites is universal, deep public health literacy is severely constrained by historical reliance on top–down, one‐way communication channels. Field data reveal that community‐wide recognition of rabies is heavily biased toward the classic “furious” clinical presentation, leaving significant vulnerabilities regarding subclinical or alternative manifestations. In the men's cohorts (FGD‐M1, FGD‐M2), participants readily identified aggressive rabid behaviors but demonstrated complete unfamiliarity with paralytic or “dumb” rabies. Conversely, women participants (FGD‐W1, FGD‐W2) frequently noted an unawareness that the virus could be transmitted via minor scratches or contact between infected saliva and broken skin. Driven by these structural knowledge gaps and a total lack of bidirectional dialogue with veterinary professionals regarding quarantine protocols, localized anxiety frequently culminates in the immediate, uncoordinated culling of suspected animals. This reactive dynamic is captured by a participant from Kiyanga Parish:

We only know about the mad dog that runs around biting people. If a dog is just quiet, weak, and hiding in the kitchen corner, we treat it as a normal sickness. Nobody from the district has ever sat down with us to explain that a quiet dog can also have the madness. (Participant 3, FGD‐M1)

This breakdown in interactive health communication extends directly into critical disconnects between theoretical medical awareness and household emergency responses. First‐line treatments for canine exposure within the sub‐county remain heavily dominated by traditional, localized practices rather than evidence‐based interventions. Women in Kashasha (FGD‐W2) noted that immediate post‐bite priorities focus on hemorrhage control and “drawing out the poison” using accessible, highly caustic substances like battery acid, chili powder, and local herbs. Crucially, the vital public health practice of immediately flushing the wound with soap and running water for 15 min was entirely absent from community narratives. Furthermore, when clinical post‐exposure prophylaxis (PEP) is sought, structural and financial barriers systematically undermine compliance. Participants from peripheral areas like Iraramira (FGD‐W1) face prohibitive motorcycle transport fares alongside chronic vaccine stockouts at public health facilities, transforming healthcare‐seeking behaviors into an uncertain economic risk:

When a child is bitten here in Iraramira, we use what we have at home—like tobacco or ash—to seal the wound. We have never received explicit instruction on using basic soap and water. Traveling to the main health center is a financial gamble because they often tell us the rabies vaccine is out of stock anyway. (Participant 7, FGD‐W1)

These systemic communication barriers directly shape how community members perceive and engage with institutional interventions. Current government‐led MDV programs are widely characterized as top–down, sporadic, and reactive, operating entirely without bidirectional planning or community mobilization. Informants in Kaburara (FGD‐M2) highlighted that vaccination campaigns are typically deployed as post hoc emergency measures following a high‐profile human rabies death in the district. Because local leaders and residents are excluded from operational planning, scheduling announcements arrive late and central distribution points are poorly placed, forcing owners to transport unrestrained, working dogs over long, hazardous distances across parish borders. To address this structural friction, an alternative approach was introduced at the vaccination site, where a novel visual signaling technique was deployed to color‐code vaccinated dogs (Figure 3). This participatory tool demonstrated how transparent, community‐facing communication can empower villagers to independently identify unvaccinated animals, fostering localized ownership and driving campaign attendance. The historical challenges of uncoordinated scheduling are illustrated by a participant from Kaburara:

**FIGURE 3:**
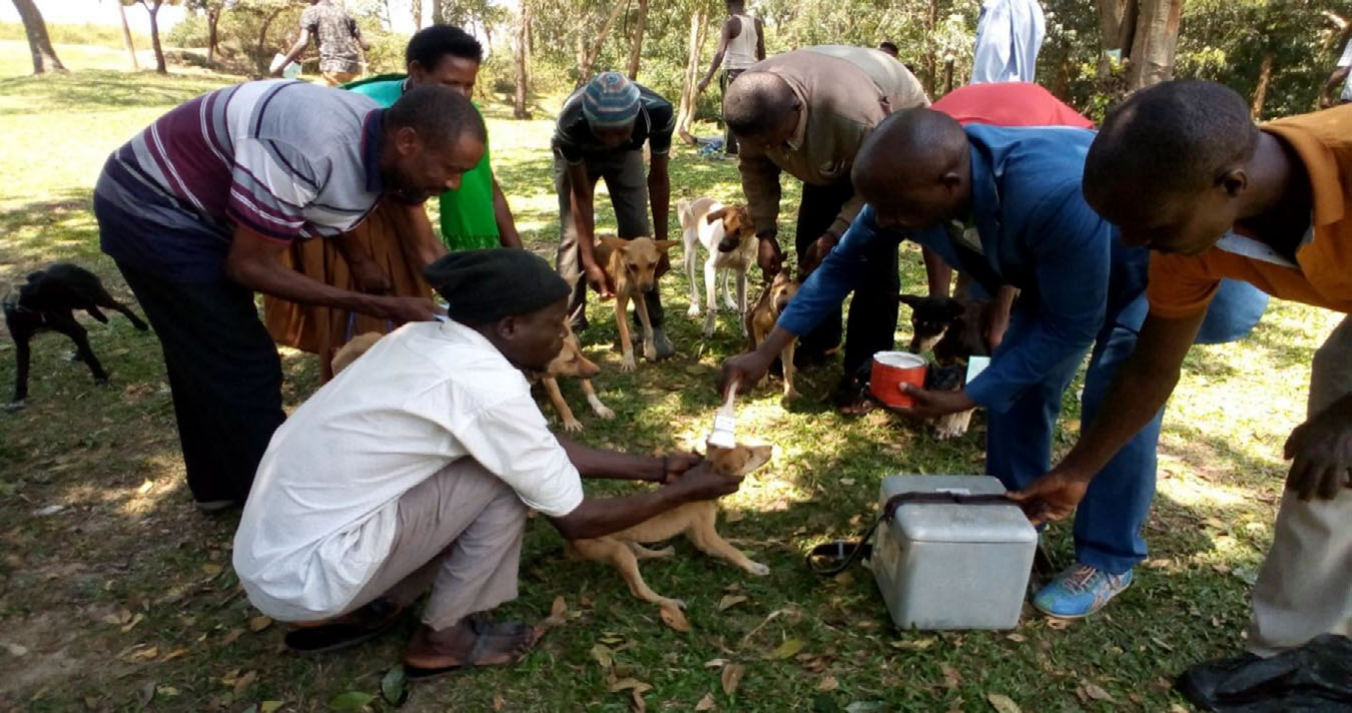
A researcher applying a temporary color marking to a dog at a mass vaccination site.

The district officials do not consult us on when or where to hold the vaccinations. They just send an announcement via word‐of‐mouth or a loudspeaker when they are already at the sub‐county headquarters. By the time you walk your dogs from the far corners of Kaburara, the officers have already left. (Participant 2, FGD‐M2)

The unique ecological landscape of Mitooma District further amplifies these public health challenges, establishing an unmonitored human–dog–wildlife interface along the edges of the Maramagambo Forest Reserve and local forest fragments. In Kiyanga Parish (FGD‐M1), participants detailed regular interactions and territorial conflicts between domestic hunting or guard dogs and wild carnivores, such as jackals and foxes. This sylvatic interface operates as a continuous, unmonitored viral reservoir for reinfection, yet it remains completely unaddressed in district veterinary outreach. In more urbanized pockets of the sub‐county, this ecological pressure is compounded by the absence of structured, humane stray dog population management. Expanding populations of free‐roaming dogs are sustained by poorly managed community garbage heaps. Deprived of institutional animal welfare support or veterinary guidance, communities frequently resort to dangerous, uncoordinated poisoning campaigns using agricultural pesticides:

Our dogs go deep into the bush for hunting and security, and they regularly fight with wild foxes and jackals. There is a continuous loop of sickness between the wild animals and our dogs, but the district veterinary office never discusses this interface with us or helps us manage it. (Participant 5, FGD‐M1)

These ecological dynamics interact with daily behavioral risks that elevate community vulnerability to subclinical transmission. Across all study sites, there is a pervasive, casual proximity to free‐roaming canines, particularly among children who regularly interact with friendly, seemingly healthy community dogs (FGD‐W1). This exposure risk is severely exacerbated by long‐term vaccine noncompliance. Although community members frequently seek initial vaccinations for puppies, annual booster compliance is virtually nonexistent due to private veterinary fees and the intense physical challenges of restraining and walking large, untrained working dogs to distant, centralized clinics:

Children pathologically trust any dog that wags its tail in the compound. They play with them daily, and we mothers only become suspicious if the dog changes its voice or starts biting objects. (Participant 1, FGD‐W1)

This systemic lack of protective infrastructure and procedural guidance ultimately disrupts critical post‐bite management protocols. The standard public health and veterinary practice of a 10‐day confinement and observation period for a biting animal is entirely absent. Driven by panic, communities almost always kill the suspected animal immediately, eliminating any opportunity for diagnostic tracking or epidemiological verification. Furthermore, even when patients successfully initiate PEP, clinical adherence remains critically deficient. Participants in Kaburara (FGD‐M2) noted that multidose compliance drops sharply once the immediate bite wound heals and localized pain subsides, as patients routinely misinterpret the resolution of visible skin trauma as a complete cure:

When the skin closes up and the pain goes away, people stop traveling to the health center. It is hard to convince someone to spend money on additional injections for an illness they can no longer see on your skin. (Participant 6, FGD‐M2)

Crucially, these behavioral patterns and public health compliance gaps are deeply anchored in the sociocultural positioning and utilitarian value of dogs within the community. Rather than being treated as companion animals, dogs are valued almost exclusively for asset security and subsistence hunting along forest borders. This functional orientation establishes distinct gender disparities that dictate campaign participation. Because these animals are raised to be fierce for security purposes, men are culturally designated as their primary owners and handlers. Women in Kashasha (FGD‐W2) explained that they and their children feel physically unempowered to restrain and walk these large, aggressive utility animals to centralized vaccination booths. Consequently, if male household heads are away during campaign hours, dogs are simply confined within the homestead, creating significant, localized gaps in community herd immunity:

A dog in this village is kept to work—either to guard the house from thieves or to chase wild animals away from our crops. Because they are raised to be fierce, it is considered the man's responsibility to handle them. If the father is not home on the day the vaccine team arrives, the family will simply lock the dog inside the compound because the women and children cannot drag a wild, heavy dog down the road. (Participant 11, FGD‐W2)

## 4 Discussion

This study demonstrates that localized community perceptions, sociocultural beliefs, and traditional medical practices present profound, deeply contextual obstacles to effective rabies post‐exposure management in Mitooma District. Specifically, our field data isolate two critical behavioral risks. These include the utilization of domestic dogs for prohibited wildlife hunting, which intensifies transmission at the sylvatic interface, and the reliance on dangerous traditional first aid, such as applying tobacco leaves, battery acid, or chili powder to bite wounds. This specific focus point, where localized ambiguity regarding clinical rabies behavior directly drives informal, nonclinical interventions, is heavily corroborated by regional literature in the Buyaga region of western Uganda [47]. Although Kankya et al. established that such ambiguity delays clinical seeking behaviors, our study extends this line of inquiry by documenting the exact home remedies used in southwestern Uganda. Globally, this structural bottleneck is further validated by studies in rural South Africa [48] and the Tanga Regional Referral Hospital in Tanzania [49], which both pinpoint traditional wound care practices as a primary barrier to immediate biomedical intervention. Consequently, these collective findings argue that rabies eradication cannot rely on top–down communication. Instead, public health frameworks must shift toward participatory health promotion that codesigns educational messages with local leaders to systematically dismantle these specific, highly localized myths.

A second critical focus point of our inquiry is the severe deficiency in community literacy regarding cross‐species transmission pathways, which directly compromises the 70% MDV threshold required for herd immunity. Our findings highlight that public health campaigns fail when they treat communities as uniform entities, ignoring how local ecology shapes awareness. This specific intersection between the ecological interface and cultural characteristics reflects broader systemic arguments raised by researchers in India [50], who demonstrated that ignoring sociocultural nuances and public awareness hurdles at the human–dog–wildlife interface inevitably dooms mass mobilization efforts. Furthermore, a national scoping review conducted across Uganda [51] explicitly pointed to the lack of community‐level operationalization of the One Health framework as a major blind spot in current research, noting that historical, purely descriptive, or human‐only studies leave substantial gaps. Our study directly addresses this empirical gap. By deploying a participatory visual signaling technique to color‐code vaccinated dogs, our field data offer a practical mechanism to bridge this knowledge divide, echoing operational successes documented during mass vaccination campaigns in rabies‐endemic regions of Haiti [52]. In that context, interactive, community‐led communication strategies successfully translated abstract epidemiological targets into actionable local knowledge, directly driving the collective compliance required to reach herd immunity targets.

By framing rabies management within a socio‐anthropological context, this study confronts a methodological bias in East African zoonotic research, which traditionally favors clinical, veterinary, or quantitative parameters while ignoring the human behavioral realities that dictate program execution. Our focus on community agency challenges the top–down models criticized in evaluations of national zoonotic health policies in Uganda [53]. These national policies argue that elimination strategies consistently falter because they treat rural populations as passive recipients of veterinary services rather than active stakeholders. The Mitooma field data provide empirical proof for this critique, demonstrating that veterinary–medical interventions are structurally limited without active community partnerships. This positions our work alongside socio‐anthropological frameworks developed in Zimbabwe [54], which assert that sustainable zoonotic disease control requires a deep understanding of community structures to identify hidden points of resistance and transform residents into active partners in disease surveillance.

Final analysis of our study underscores that MDV coverage is not merely a logistical function of vaccine supply but is heavily constrained by an intersecting matrix of historical, social, political, economic, and ecological contextual factors. Our focus on operational friction, specifically late announcements, poor distribution geometry, and the physical challenges of restraining large working dogs, is strongly supported by empirical evidence from Kyegegwa District, Uganda [55]. Prior findings in Kyegegwa isolated geographic terrain, deep‐seated community dynamics, severe economic constraints, and systemic shortages of veterinary personnel as compounding barriers to campaign optimization. Similar multi‐factorial vulnerabilities were documented in Tanzania [56], confirming that isolated vaccine drops are insufficient and that addressing these overlapping vulnerabilities requires moving away from uncoordinated interventions. Our study advances this discussion by presenting participatory communication as a direct operational solution to these barriers. Involving local leaders in campaign logistics allows communities to co‐map vaccination boundaries around farming schedules and geographical hurdles, transforming responsible dog ownership from an abstract local demand into a systemically supported reality.

Improving local participation requires a comprehensive overhaul of how responsible dog ownership is conceptualized and supported both at the community and systemic levels. Responsible ownership, encompassing canine confinement, adequate nutrition, and routine vaccination, cannot be sustained through localized messaging alone if systemic institutional support remains absent. This dual requirement for local accountability and systemic infrastructure aligns with prior research demonstrating that participatory communication fosters a complementary dual awareness [57]. Locally, it shifts community perceptions of dogs from loose, utilitarian assets to protected domestic animals; systemically, it leverages community‐generated data to advocate for consistent veterinary infrastructure, ensuring that community willingness is effectively matched by institutional capacity. Because structural vaccine compliance and dog bite incidences are dynamically and heavily correlated [58], utilizing participatory communication to optimize localized immunization infrastructure serves as an immediate, practical prerequisite to achieving the World Health Organization global strategic plan to eliminate dog‐mediated human rabies deaths by 2030 (WHO “Zero by 30”).

## 5 Conclusion and Recommendation

This exploratory study demonstrates that the persistence of domestic rabies in Mitooma District is not merely a consequence of vaccine scarcity, but rather a fundamental failure of community engagement and participatory communication. Historical reliance on top–down, instructional public health models has left deep public literacy deficits, sustained hazardous traditional wound care practices, and resulted in fragmented, low‐coverage MDV campaigns. Furthermore, the unmonitored human, domestic dog, and wildlife interface in ecological hotspots like Kiyanga Parish creates a continuous epidemiological threat that standard, reactive public health tactics cannot contain. Achieving sustainable zoonotic disease elimination requires that the Mitooma District Local Government operationalize a functional One Health framework driven by participatory communication, textually transforming local communities from passive, fearful onlookers into active, accountable co‐managers of public and animal health.

To translate these empirical insights into actionable interventions, the Mitooma District Local Government must immediately formalize a joint multi‐sectoral taskforce integrating the District Health Officer, the District Veterinary Officer, environmental authorities, and representatives from the Uganda Wildlife Authority. Meeting quarterly, this intersectoral body can synchronize human and animal disease surveillance, co‐budget mass canine vaccination campaigns, and mitigate spillover risks at the forest and agriculture frontier. Concurrently, district veterinary authorities must abandon top–down administrative scheduling models and work directly with local Parish Development Committees and Local Council I chairpersons to co‐select decentralized vaccination stations, establish optimal seasonal timing aligned with community farming calendars, and leverage local information paths to maximize community turnout. Local authorities should also transform rabies communication by training Village Health Teams to deliver interactive, community dialogue sessions rather than relying on passive print media. These educational campaigns must move away from high‐fear imagery and focus on actionable knowledge, including recognizing the dumb paralytic form of rabies, practicing proper 15 min soap and water wound washing, and emphasizing the lifesaving necessity of complete multidose PEP compliance.

Finally, long‐term stabilization requires sub‐county and district councils to draft, gazette, and actively enforce localized animal control bylaws. These regulations must mandate the registration of domestic canines, establish clear penalties for owners who abandon or refuse to confine utility dogs at night, and formalize legal mechanisms to hold owners financially liable for livestock damage or human bite medical costs. To complement these legal guardrails, the district should train local hunters, farmers, and school teachers to serve as community surveillance focal points. Utilizing simple, mobile‐based reporting systems or dedicated local communication channels, these individuals can report animal bites, stray packs, or unusual wildlife encounters directly to the One Health taskforce, enabling rapid, preventive field interventions before human exposures occur. Through this integrated approach, the district can bridge immediate operational gaps and actively contribute toward the realization of global disease elimination targets.

## Author Contributions

**Augustino Ngabirano**: conceptualization, investigation, writing – original draft, methodology, writing – review and editing, project administration, data curation, resources. **Matthew Chibunna Igwe**: methodology, validation, formal analysis, supervision, data curation, writing – review and editing. **Charles Idehen**: data curation, supervision, formal analysis, methodology, validation, writing – review and editing. All authors read and approved the final manuscript.

## Funding

The authors have nothing to report.

## Ethics Statement

This study was approved as an institutional program evaluation by the Mitooma District Local Government and conducted under the lead investigator's Kampala International University doctoral research protocols.

## Consent

Informed consent was obtained from all participants involved in the exploratory study.

## Conflicts of Interest

The authors declare no conflicts of interest.

## Data Availability

The qualitative and spatial data supporting the findings of this study are available from the corresponding author upon reasonable request.
